# Thermoelectric Properties of Hot-Pressed Bi-Doped n-Type Polycrystalline SnSe

**DOI:** 10.1186/s11671-018-2500-y

**Published:** 2018-07-06

**Authors:** Van Quang Nguyen, Thi Huong Nguyen, Van Thiet Duong, Ji Eun Lee, Su-Dong Park, Jae Yong Song, Hyun-Min Park, Anh Tuan Duong, Sunglae Cho

**Affiliations:** 1 Department of Physics and Energy Harvest Storage Research Center, University of Ulsan, Ulsan, 44610 Republic of Korea; 2 Thermoelectric Conversion Research Center, Creative and Fundamental Research Division, Korea Electrotechnology Research Institute (KERI), Changwon, 51543 Republic of Korea; 3 Materials Genome Center, Korea Research Institute of Standards and Science, Daejeon, 305-340 Republic of Korea; 4 Phenikaa Research and Technology Institute, A&A Green Phoenix Group, 167 Hoang Ngan, Hanoi, 10000 Vietnam

**Keywords:** Thermoelectricity, 2D materials, SnSe, Hot press, Bipolar transport

## Abstract

**ᅟ:**

We report on the successful preparation of Bi-doped n-type polycrystalline SnSe by hot-press method. We observed anisotropic transport properties due to the (h00) preferred orientation of grains along the pressing direction. The electrical conductivity perpendicular to the pressing direction is higher than that parallel to the pressing direction, 12.85 and 6.46 S cm^−1^ at 773 K for SnSe:Bi 8% sample, respectively, while thermal conductivity perpendicular to the pressing direction is higher than that parallel to the pressing direction, 0.81 and 0.60 W m^−1^ K^−1^ at 773 K for SnSe:Bi 8% sample, respectively. We observed a bipolar conducting mechanism in our samples leading to n- to p-type transition, whose transition temperature increases with Bi concentration. Our work addressed a possibility to dope polycrystalline SnSe by a hot-pressing process, which may be applied to module applications.

**Highlights:**

1. We have successfully achieved Bi-doped n-type polycrystalline SnSe by the hot-press method.
2. We observed anisotropic transport properties due to the [h00] preferred orientation of grains along pressing direction.
3. We observed a bipolar conducting mechanism in our samples leading to n- to p-type transition.

**Electronic supplementary material:**

The online version of this article (10.1186/s11671-018-2500-y) contains supplementary material, which is available to authorized users.

## Background

Thermoelectric materials can directly convert waste heat into electricity, which is one of the most important global sustainable energy solutions, or can be used as solid-state Peltier coolers. These thermoelectric devices have exhibited many advantages such as no involvement of moving part, small size, light weight, no noise, no pollution, and long life service. However, their applications are still limited by the economical reasons and low energy conversion efficiency, which is evaluated by the dimensionless thermoelectric figure of merit, ZT = *S*^2^*σT*/*κ*, where *S* is the Seebeck coefficient, *T* is absolute temperature, *σ* is electrical conductivity, and *κ* is thermal conductivity. The good thermoelectric material should have a high Seebeck coefficient, high electrical conductivity, and low thermal conductivity. However, these three transport coefficients are inter-dependent [1]. There are two main ways to enhance ZT, enhancing power factor (PF, *S*^2^*σ*) or lowering total thermal conductivity. Electrical conductivity and Seebeck coefficient are inversely related each other in most materials, which limit the thermoelectric power factor. Lower thermal conductivity can be achieved by increasing the phonon scattering center or adding a number of interfaces in materials such as superlattices, alloys, nanowires, and nanotubes. Bi_2_Te_3_ and PbTe are two traditional thermoelectric materials, whose ZTs are much improved, 1.8 at 320 K for Bi_0.5_Sb_1.5_Te_3_ [2] and 2.2 at 915 K for PbTe + 2%Na + 4%SrTe [3]. However, there are many disadvantages for the systems because Bi and Te elements are rare on the earth, resulting in increase of costs with the development of the LED industry [4], and lead is a toxic element. Therefore, it is necessary to explore economical and non-toxic (lead-free) alternative materials for thermoelectric applications.

IV–VI compound semiconductor SnSe is a robust candidate for thermoelectric conversion applications, which has been recently reported with high thermoelectric performance, ZT = 2.6 at 923 K in un-doped p-type and ZT = 2.0 at 773 K in intentionally hole-doped SnSe single crystal [5, 6]. Recently, we achieved ZT = 2.2 in n-type Bi-doped SnSe single crystal [7]. These high ZT values are attributed to the ultralow intrinsic thermal conductivity due to the long-range interaction along the <100> direction caused by resonant bonding, leading to optical phonon softening, strong anharmonic scattering and large phase space for three-phonon scattering processes [8]. Bulk SnSe belongs to orthorhombic *Pnma* space group (*a* = 11.49 Å, *b* = 4.44 Å, *c* = 4.14 Å) with an indirect band gap energy of *E*_g_ = 0.829 eV at 300 K. When temperature is increased, it changes to orthorhombic *Cmcm* space group (*a* = 11.71, *b* = 4.31, and *c* = 4.32 Å) with a direct band gap of *E*_g_ = 0.464 eV around 807 K [9]. SnSe exhibits a two-dimensional (2D) layered structure, where each Sn atom is surrounded by a highly distorted octahedron of Se atoms to form a zigzag structure. Along the *b*-*c* plane, there is a strong Sn–Se covalent bonding, and along the *a*-axis, there is a weak van der Waals force, which gives a strong anisotropic transport and very weak mechanical properties. The most common technique to fabricate single-crystal SnSe is the Bridgman technique which is quite specific and hard to produce in industry scale-up [1]. Considering the large-scale applications and the poor mechanical properties in layered material, polycrystalline SnSe is a possible solution.

Recently, un-doped p-type polycrystalline SnSe has been reported with ZT = 0.5 at 823 K and ZT = 1.3 at 850 K for rock-salt SnSe, and doped p-type SnSe has been reported with the highest ZT = 0.6 at 750 K for Ag dopant [1, 10, 11]. Polycrystalline n-type SnSe has been reported with the ZT range from 0.6 to 1.2 for Te, I, BiCl_3_, and Br dopants [4, 12–14]. Hot pressing and spark plasma sintering (SPS) are the most general techniques used to fabricate a polycrystalline of un-doped and doped SnSe.

Here we report on the successful preparation of Bi-doped n-type polycrystalline SnSe by hot-press method. We observed anisotropic transport properties due to the (h00) preferred orientation of grains along pressing direction. We also observed a bipolar conducting mechanism in our samples leading to n- to p-type transition, whose transition temperature increases with Bi concentration.

## Methods/Experimental

The aim of this paper is fabricating and investigating thermoelectric properties of n-type Bi-doped SnSe polycrystalline with various Bi concentrations (0, 2, 4, 6, and 8%). The doping process is fulfilled by mixing and hot-pressing SnSe with Bi powders. The details of fabrications and characterizations of the samples are as below.

### Fabrication of SnSe Compound by Temperature Gradient Technique

We fabricated the SnSe compound using the temperature gradient technique. The high-purity (99.999%) Sn and Se powders were weighed in an atomic ratio of 1:1 using a balance with a resolution of 10^−4^ g. The powders were mixed and sealed in an evacuated (< 10^−4^ Torr) quartz ampoule. The ampoule was then sealed in another evacuated bigger quartz ampoule in order to prevent the sample from oxidation by air in the case when the inner ampoule is broken owing to the difference of thermal expansion between the crystal and quartz. The ampoules were slowly heated up to 600 °C for 30 h. It was maintained at this temperature for 1 h and then continuously heated up to 950 °C for 35 h. To complete the reaction between Sn and Se, we maintained the ampoules at this temperature for 16 h and then slowly cooled down to room temperature. An excellent SnSe compound with dimensions of 13 mm diameter × 25 mm length was obtained.

### Fabrication of n-Type Bi-Doped SnSe Polycrystalline Samples by Hot-Press Technique

The obtained ingots above were ground into powders and mixed with various Bi (0, 2, 4, 6, and 8%) amounts for 1 h using a mixing machine. The mixed powder was loaded into a 13-mm diameter mold and then hot-pressed at 800 °C using 30 MPa pressure in Ar environment for 30 min to form a dense pellet with a 13-mm diameter and 15-mm length.

### Characterizations

The samples were analyzed by X-ray diffraction (XRD) both parallel and perpendicular to the pressing direction. Field emission scanning electron microscopy (FE-SEM) was used to observe the microscopic image in the fractured surface of the samples. To probe the anisotropic transport and thermoelectric properties, the samples were cut into 2 × 1.5 × 8 mm bars for transport and 13 × 13 × 1.5 mm for thermal diffusivity measurements along both parallel (//) and perpendicular (⊥) directions using a diamond saw. Electrical conductivity and the Seebeck coefficient were simultaneously collected from room temperature to 773 K with a collinear four-probe configuration under an Ar atmosphere to prevent oxidation and evaporation of sample. The laser flash diffusivity method (model: LFA-457, NETZSCH, Germany) was used to determine thermal diffusivity from room temperature to 773 K. Mass density was determined by measuring the sample’s dimensions and mass. Heat capacity was taken from Sassi’s work for polycrystalline SnSe [1]. Thermal conductivity was calculated by the relationship *κ = DC*_*p*_*ρ*, where *D*, *C*_*p*_, and *ρ* are the thermal diffusivity, the heat capacity, and the mass density, respectively.

## Results and Discussion

**Fig. 1:**
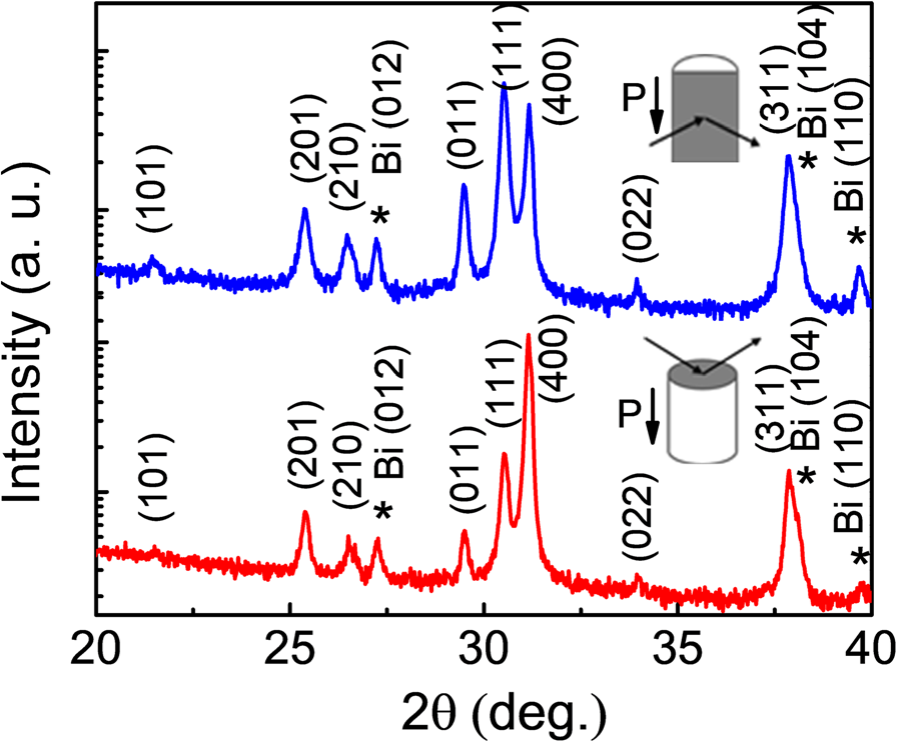
(Color online) Room temperature XRD patterns for SnSe:Bi 4% perpendicular (red color) and parallel (blue color) to the pressing direction as illustrated in the inset. The figure showed the orthorhombic structure and the presence of rhombohedral Bi phase

The room temperature XRD patterns of sample SnSe:Bi 4% in both ⊥ and // directions are shown in Fig. 1, which are indexed based on the orthorhombic SnSe phase (space group *Pnma*). In the patterns, there are several small peaks, which are identified as rhombohedral Bi. This dominant Bi secondary phase indicates that SnSe does not decompose at 800 °C and other phases such as BiSnSe or Bi_2_Se_3_ are not formed. The average lattice parameters estimated from XRD patterns were *a* = 11.469, *b* = 4.143, and *c* = 4.435 Å, in good agreement with the previous reports [1, 4]. The patterns also showed strong (400) peak intensities in the plane parallel to the pressing direction, indicating that grains have preferentially aligned along the [h00] direction due to the layered structure of SnSe.

**Fig. 2:**
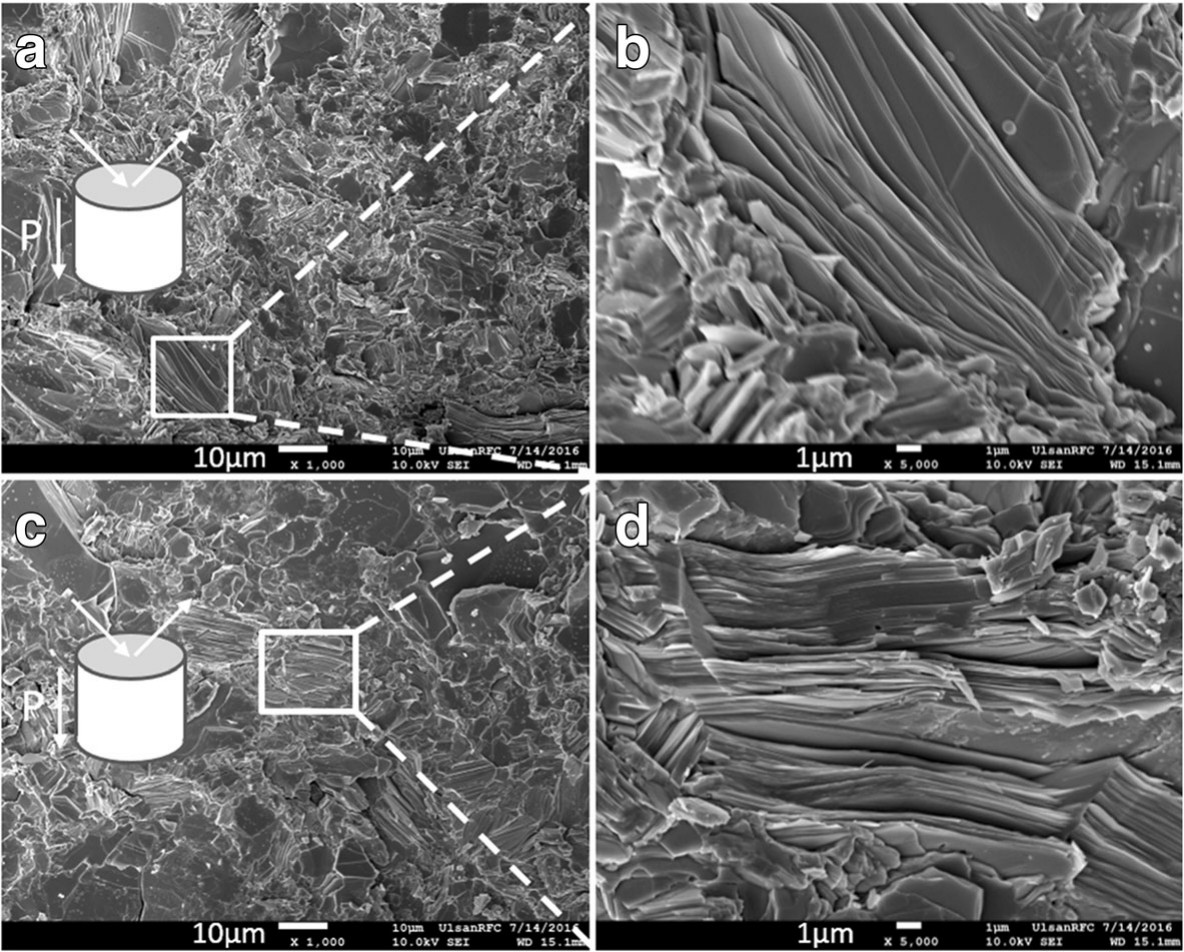
FE-SEM images of the fractured surfaces along the ⊥ direction of sample SnSe:Bi 4% (**a**, **b**) and SnSe:Bi 6% (**c**, **d**). FE-SEM images showed the layered structure and the dominant layers on the plane perpendicular to the pressing direction

The surface SEM images of the fractured SnSe:Bi 4% (a, b) and SnSe:Bi 6% (c, d) samples are shown in Fig. 2, which were taken on the plane parallel to the pressing direction as defined in Fig. 2. As shown in the figure, our samples exhibited the layered structure with the fragments of layers tending to lie on the plane. Some tilted layers were seen in Fig. 2b, c. On the other hand, when the Bi doping content increased from 4 to 6%, the estimated grain size increased from 3 to 10 μm. This observation indicated that Bi was not only substituted for Sn but also played as a flux leading to the increase in grain size.

**Fig. 3:**
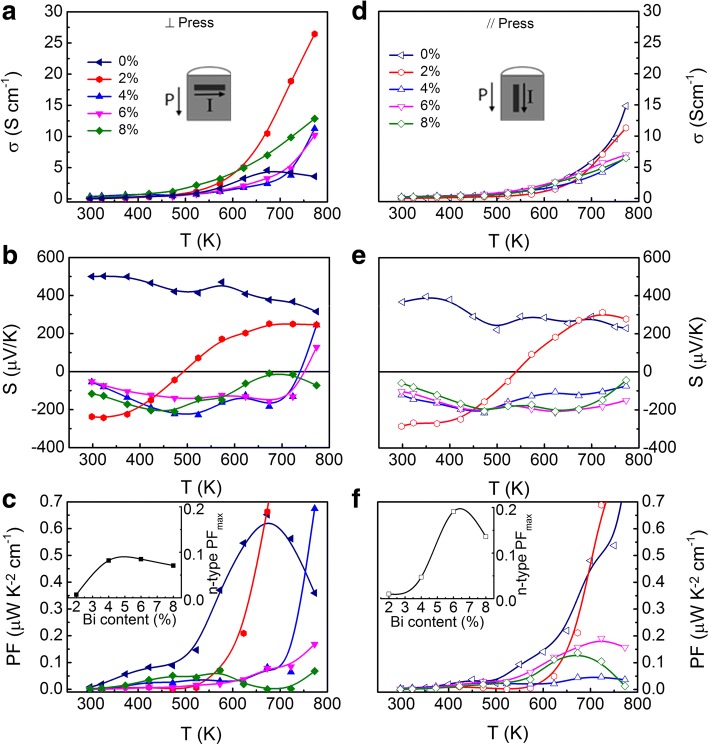
(Color online) Temperature dependence of the electrical conductivity (**a**, **d**), Seebeck coefficient (**b**, **e**), and power factor (**c**, **f**) of samples with various Bi contents along ⊥ and // directions as defined in the inset of **a** and **d**, where the black arrows indicated the press direction P. The n-type maximum power factor as a function of Bi content is shown in the inset of **c** and **f**

The temperature-dependent Seebeck coefficient (S), electrical conductivity, and power factor of samples for ⊥ and // directions are shown in Fig. 3. The figure showed the anisotropic transport properties, which are dependent on pressing directions. The electrical conductivity along the ⊥ direction is higher than that along the // direction due to the preferred orientation of the hot-pressed sample as mentioned above. Considering n-type samples, along the ⊥ direction, the electrical conductivity increased with Bi content, while along the // direction, it reached the maximum value in the SnSe:Bi 6% sample and then decreased in the SnSe:Bi 8% sample. The electrical conductivities in all samples along both directions rise with temperature, indicating a typical semiconductor behavior as shown in Fig. 3a, d. There was no metallic behavior above 700 K in our data, which is different from the previous reports due to the re-evaporation of Se at high temperature [1, 13]. This behavior confirmed the stability of our samples with the measured temperature range under an Ar atmosphere.

A small anisotropy in the Seebeck coefficient was observed as shown in Fig. 3b, e. Positive Seebeck coefficient was observed in the un-doped sample, while negative Seebeck coefficients were observed in Bi-doped samples, indicating the substitution of Bi into the Sn site. The temperature-dependent Seebeck coefficient curves of Bi-doped samples showed n- to p-type transitions. Along the ⊥ direction, the transition temperatures were 492, 730, and 762 K for SnSe:Bi 2, 4, and 6% samples, respectively, while no transition was observed for SnSe:Bi 8% sample. Along the // direction, the transition was observed at 541 K only for SnSe:Bi 2% sample. The absence of the n- to p-type transitions in some samples may be due to higher transition temperatures than our maximum measured temperature, 773 K. These n- to p-type transitions are related to the bipolar conducting mechanism in our samples. The substituted Bi provided electrons to the conduction band and the Sn vacancies, played as the acceptors, and generated holes in the valence band. As the temperature increases from 300 K, the donor impurities are activated and then n-type conduction is dominant. As a result, negative *S* is achieved. When the temperature is above a critical point, electrons in the valence band gain enough thermal energy to elevate to the acceptor levels and then holes are generated. When the hole becomes a dominant charge carrier, positive *S* is achieved. The contribution of the electrons and the holes to *S* compensated each other and decreased *S*. Since, *S* can be calculated by the following formula for semiconductor:

1 $$ S=\frac{p{\mu}_p{S}_p-n{\mu}_n{S}_n}{n{\mu}_n+p{\mu}_p}\kern1.25em $$

where *S* is the total Seebeck coefficient, *n* and *p* are the electron and the hole concentrations, *μ*_*p*_ and *μ*_*n*_ are the electron and the hole mobility, and *S*_*p*_ and *S*_*n*_ are contributions of the electron and the hole to *S*. As shown in Fig. 3b, e, the n- to p-type transition temperature along the ⊥ direction is lower than that along the // direction. This observation can be easily understood due to the higher electrical conductivity, indicating the higher mobility of charge carriers along the ⊥ direction than that along the // direction. As shown in Fig. 3a, b, d, and e, below the transition temperature, the electron carrier is dominant and its mobility along the ⊥ direction is larger than that of electron along the // direction. However, above the transition, the hole carrier is dominant with much higher hole mobility along the ⊥ direction. Thus, the Seebeck coefficient transition along the ⊥ direction occurs first. This transition temperature also increases with Bi content, indicating the substitution of Bi for Sn in SnSe lattice. As a result of the small Seebeck coefficient and electrical conductivity, very small power factor values are achieved (Fig. 3c, f). The inset of Fig. 3c, f shows maximum power factors of n-type samples as a function of the Bi content. These values of power factor are higher along the // direction than those along the ⊥ direction The power factor reached a maximum value of 0.19 μW/cm K^2^ in SnSe:Bi 6% sample along the // direction.

**Fig. 4:**
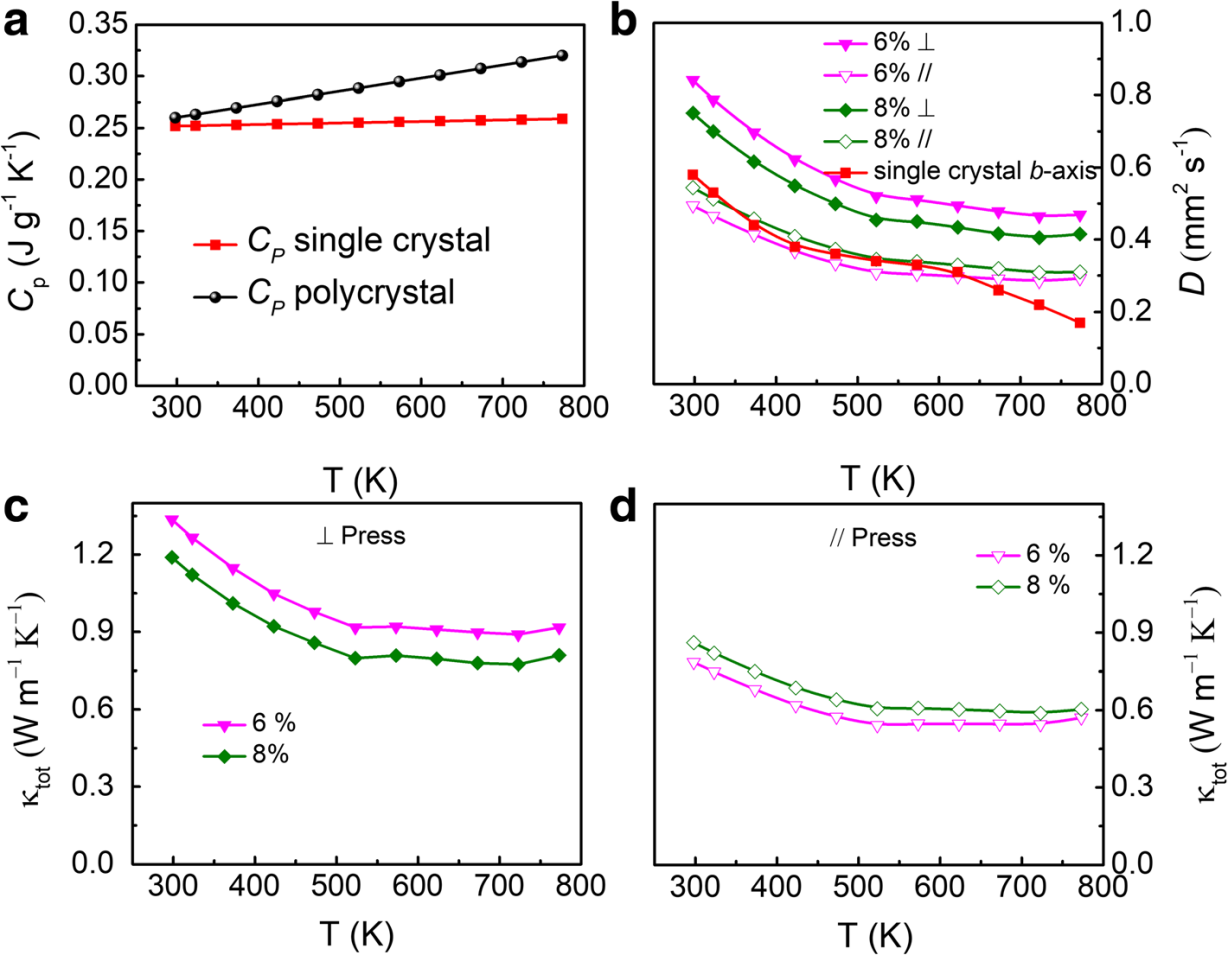
(Color online) Temperature dependence of heat capacity (*C*_*p*_) taken from [1] (**a**), thermal diffusivity (*D*) (**b**), and thermal conductivity (*κ*) of SnSe:Bi 6% and SnSe:Bi 8% samples along both ⊥ and // directions compared with Bi-doped n-type SnSe single crystal [7] (**c**, **d**)

Figure 4 shows the temperature dependence of heat capacity (*C*_*p*_), thermal diffusivity (*D*), and thermal conductivity (*κ*) of polycrystalline SnSe:Bi 6% and SnSe:Bi 8% samples along both directions, which exhibited higher power factors. The lowest thermal conductivity of 0.544 W/m K is obtained along the // direction at 723 K SnSe:Bi 6% sample (Fig. 4c). The thermal conductivities along both directions are comparable with other reports for polycrystalline SnSe [1, 9–13] and lower than that of single-crystal SnSe:Na [6]. However, these values are higher than that of un-doped p-type SnSe [5] and Bi-doped n-type SnSe single crystal [7]. Note that the thermal conductivity is proportional to mass density, heat capacity, and thermal diffusivity of the material. Polycrystalline samples are expected to have similar or even lower thermal conductivity values due to the additional phonon scatterings by grain boundaries. One possible reason for this high thermal conductivity was suggested by Zhao et al. [6] as the surface oxidation of samples due to the air exposure. However, Ibrahim et al. [15] disagreed with this opinion. Another reason for the high thermal conductivity is micro-crack in the samples, which may double the thermal conductivity of SnSe suggested by Zhao et al. [16]. This micro-crack may come from the cutting and polishing processes during the sample preparation for thermal diffusivity measurements. In this work, we took the heat capacity values from Sassi’s work [1] for polycrystalline SnSe, which are higher than that for single crystalline SnSe in [5, 7] as shown in Fig. 4a. Note that we have linearly extrapolated Sassi’s temperature-dependent heat capacity from 300 to 773 K. The measured thermal diffusivity was higher along the perpendicular and comparable along the parallel direction compared to that along the *b*-axis for Bi-doped n-type SnSe single crystal (Fig. 4b). The mass densities were comparable with n-type Bi-doped SnSe single crystalline samples [7], 6.11 and 6.09 g/cm^−3^ for samples SnSe:Bi 6% and 8%, respectively. Therefore, we conclude that the higher thermal conductivity in our polycrystalline samples than that in single crystalline samples comes from the higher values of thermal diffusivity and specific heat.

**Fig. 5:**
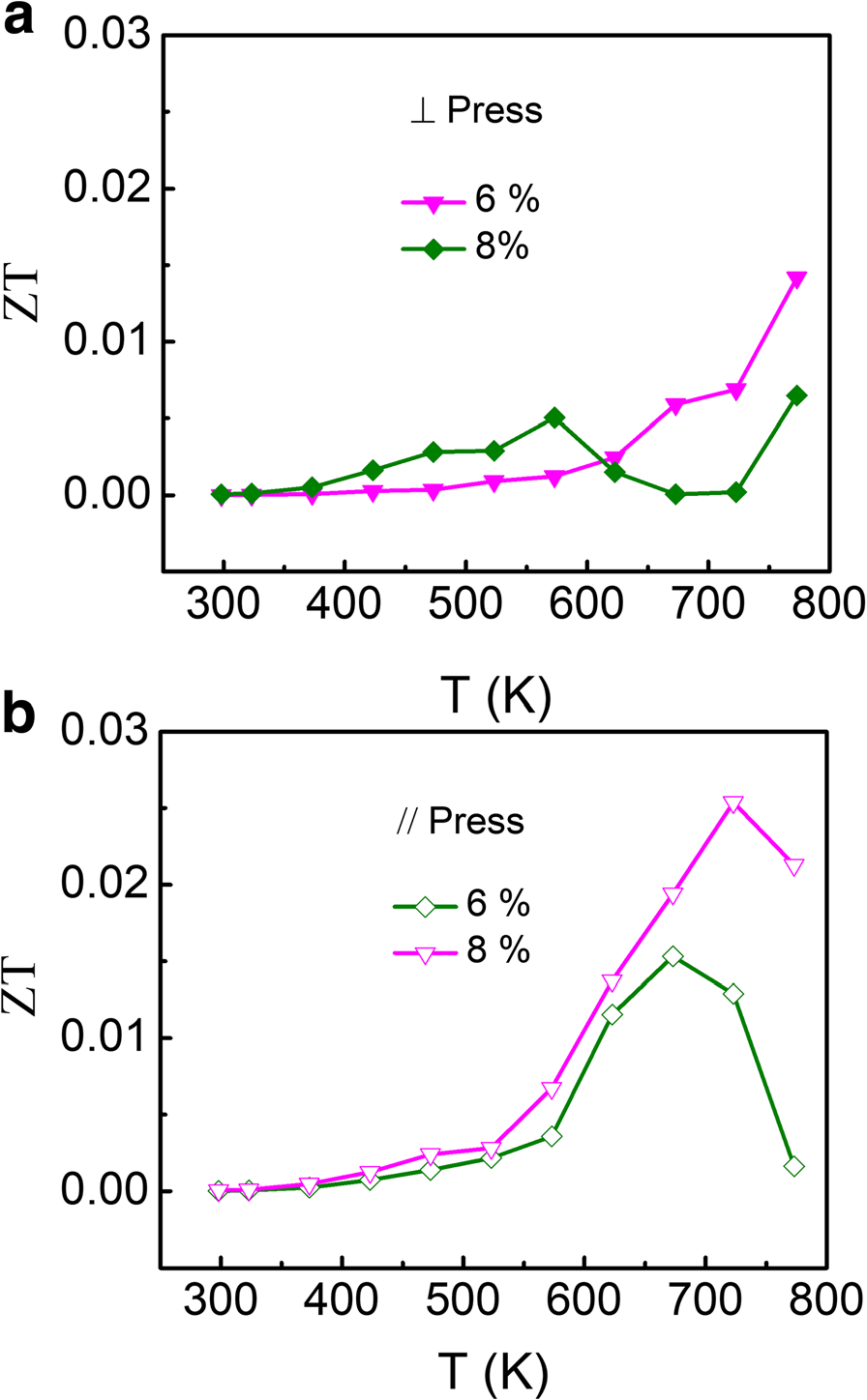
(Color online) Temperature dependence of dimensionless thermoelectric figure of merit of polycrystalline SnSe:Bi 6% and SnSe:Bi 8% samples along both ⊥ (**a**) and // (**b**) directions

The dimensionless figure of merit ZT values as a function of temperature for these samples along both directions are shown in Fig. 5. The highest ZT of 0.025 is obtained at 723 K along the // direction for SnSe:Bi 6% sample, which seems to be the optimal doping content. Due to the small electrical conductivity, the total thermal conductivity is mostly attributed to the lattice thermal conductivity. Therefore, lower thermal conductivity is obtained along the // direction owing to the weak atomic connections. Consequently, higher ZT values are obtained along the // direction. However, these ZT values are quite small compared to those of single crystal or even other polycrystalline SnSe due to the lower *S* and *σ* values.

## Conclusions

In conclusion, polycrystalline SnSe has been doped with various Bi concentrations by hot-press method (Additional file 1). The samples exhibited the layered structure with a preferential (h00) orientation. An anisotropic transport and thermoelectric properties have been observed. The electrical conductivities perpendicular to the pressing direction (12.85 S cm^−1^) are higher than those parallel to the pressing direction (6.46 S cm^−1^) at 773 K for SnSe:Bi 8% sample, while thermal conductivities perpendicular to the pressing direction (0.81 W m^− 1^ K^−1^) are higher than those parallel to the pressing direction (0.60 W m^−1^ K^−1^) at 773 K for SnSe:Bi 8% sample. We observed a bipolar conducting mechanism in our samples leading to n- to p-type transition, whose temperature increases with Bi concentration. The optimum Bi doping concentration was 6% with the highest ZT value of 0.025 at 723 K. This ZT value is quite low due to the low electrical conductivity and Seebeck coefficient. Our work addressed a possibility to dope polycrystalline SnSe by a hot-pressing process, which may be applied to module applications.

## Additional file

Additional file 1: Excel source data. (XLSX 196 kb)

## Abbreviations

//: Parallel
⊥: Perpendicular
*C*_*p*_: Specific heat
*D*: Thermal diffusivity
FE-SEM: Field emission scanning electron microscopy
PFs: Power factors
*S*: Seebeck coefficient
*T*_max_: Maximum temperature
XRD: X-ray diffraction
ZT: Thermoelectric figure of merit
*κ*: Thermal conductivity
*μ*_*n*_: Electron mobility
*μ*_*p*_: Hole mobility
*ρ*: Mass density
*σ*: Electrical conductivity
